# Terrestrial invasive species alter marine vertebrate behaviour

**DOI:** 10.1038/s41559-022-01931-8

**Published:** 2023-01-05

**Authors:** Rachel L. Gunn, Cassandra E. Benkwitt, Nicholas A. J. Graham, Ian R. Hartley, Adam C. Algar, Sally A. Keith

**Affiliations:** 1 Lancaster Environment Centre, Lancaster University, Lancaster, UK; 2 Department of Biology, Lakehead University, Thunder Bay, Ontario Canada

**Keywords:** Behavioural ecology, Invasive species, Stable isotope analysis

## Abstract

Human-induced environmental changes, such as the introduction of invasive species, are driving declines in the movement of nutrients across ecosystems with negative consequences for ecosystem function. Declines in nutrient inputs could thus have knock-on effects at higher trophic levels and broader ecological scales, yet these interconnections remain relatively unknown. Here we show that a terrestrial invasive species (black rats, *Rattus rattus*) disrupts a nutrient pathway provided by seabirds, ultimately altering the territorial behaviour of coral reef fish. In a replicated ecosystem-scale natural experiment, we found that reef fish territories were larger and the time invested in aggression lower on reefs adjacent to rat-infested islands compared with rat-free islands. This response reflected changes in the economic defendability of lower-quality resources, with reef fish obtaining less nutritional gain per unit foraging effort adjacent to rat-infested islands with low seabird populations. These results provide a novel insight into how the disruption of nutrient flows by invasive species can affect variation in territorial behaviour. Rat eradication as a conservation strategy therefore has the potential to restore species interactions via territoriality, which can scale up to influence populations and communities at higher ecological levels.

## Abstract (web summary)

In a replicated ecosystem-scale natural experiment across ten islands in the Indian Ocean, invasive black rats disrupted nutrients provided by seabirds, leading to a coral reef fish having larger territories and investing less time in aggression than on rat-free islands.

## Main

The movement of naturally occurring nutrients across habitats and ecosystems is a strong driver of productivity and can influence community dynamics^1^. Inputs from animals can contribute substantially to the nutrient budget of an ecosystem, but anthropogenic activities have reduced the movement of naturally occurring nutrients between animals to 6% of historic values^2,3^. While certain aspects of human-induced environmental change can increase the nutrient load to an ecosystem^4^, human-induced declines in the movement of nutrients can negatively impact the resources that organisms are able to exploit^2^. Organisms initially respond to human-induced environmental change through rapid behavioural modifications that reduce the resource demand of the organism, including changes to foraging behaviour^5,6^, aggression^7,8^ and territoriality^9^. Thus, reductions in nutrient inputs are predicted to alter the behaviour of higher-trophic-level organisms via cascading effects through food webs, although such connections are untested. As changes in behaviour can influence species interactions and subsequently shape ecological communities^10,11^, understanding behavioural responses to change is fundamental to revealing the complex ecological consequences of human-induced nutritional declines.

Nutritional resources are key drivers of territorial behaviour. The food maintenance hypothesis predicts that territory size is primarily determined by the nutritional requirements that allow organisms to meet short-term energetic needs^12^. An inverse relationship between resource availability and territory size^13^ is apparent across a range of organisms including mammals^14^, reptiles^15^, birds^16^ and fish^17^. Theory proposes that aggression is also largely determined by nutritional resources, such that the aggressive defence of territories, or territoriality, is predicted to occur only where the energetic benefits of territoriality outweigh the costs^18^. Under this model of economic defendability, a bell-shaped relationship between territoriality and resource level is predicted, and territoriality is only beneficial when the value of a nutritional resource is above a certain threshold value^19^. When nutritional resources are rare, the energetic cost of territoriality is too high. At intermediate resource levels, positive net benefits for territoriality outweigh the benefits of non-territorial behaviour, and the net pay-off for territorial behaviour reaches a peak^18,20^. Where resources are in excess, the highest net benefit occurs for non-territorial behaviour, as individuals can obtain resources at low cost without the need for aggression^19^. It is plausible that the disruption of nutrient pathways by human-induced environmental change lowers the value of nutritional resources, impacting the cost-benefit dynamics of territoriality and the subsequent territorial tendencies of individuals^13,19^. However, the consequences of declines in nutrient transfer on variation in territorial behaviour remains untested.

Seabirds are globally important contributors to nutrient transfer, responsible for a cascade of nutrients through terrestrial^21^ and marine^22^ ecosystems by depositing guano on islands after feeding in the open ocean. Invasive species, including black rats (*Rattus rattus*), disrupt this nutrient pathway by driving declines in seabird densities via predation^23^. Nutrient subsidies from seabirds flowing onto coral reef ecosystems result in higher nitrogen stable isotope quantities (δ^15^N) in algae and fish^24,25^, enhanced coral growth^26^, higher reef fish biomass^27^ and faster growth rate in herbivorous fishes^22,28^. Furthermore, the presence of invasive rats negatively impacts reef fish diversity and ecosystem function on adjacent coral reefs^29^. Invasive rats, therefore, have a substantial bottom-up impact on terrestrial and marine ecosystems. As behaviour is an important mediator between organisms and the environment^11^, the behavioural responses of reef organisms could play a mechanistic role in the ecological changes observed on coral reefs adjacent to rat-infested islands.

Here we use a control-treatment experimental design to provide a novel insight into the cascading effects of seabird nutrient subsidies on territorial behaviour and reveal how these effects are disrupted by invasive rats, using the herbivorous farmer damselfish, *Plectroglyphidodon lacrymatus*. We test the model of economic defendability^18^ and identify the role of nutritional subsidies in placing the value of resources above the critical threshold value for territoriality. We predict that the nutrient enrichment of resources by seabirds will result in higher aggression and a smaller territory size for *P. lacrymatus* individuals on reefs around rat-free islands compared with individuals on reefs around rat-infested islands. Using in situ observations, we link the aggression of individual fish to their territory size, and the quantity and nitrogen enrichment of their nutritional resources to show how a reduction in nutrient subsidies can drive both broad and fine-scale variability in territorial behaviour.

**Extended Data Fig. 1:**
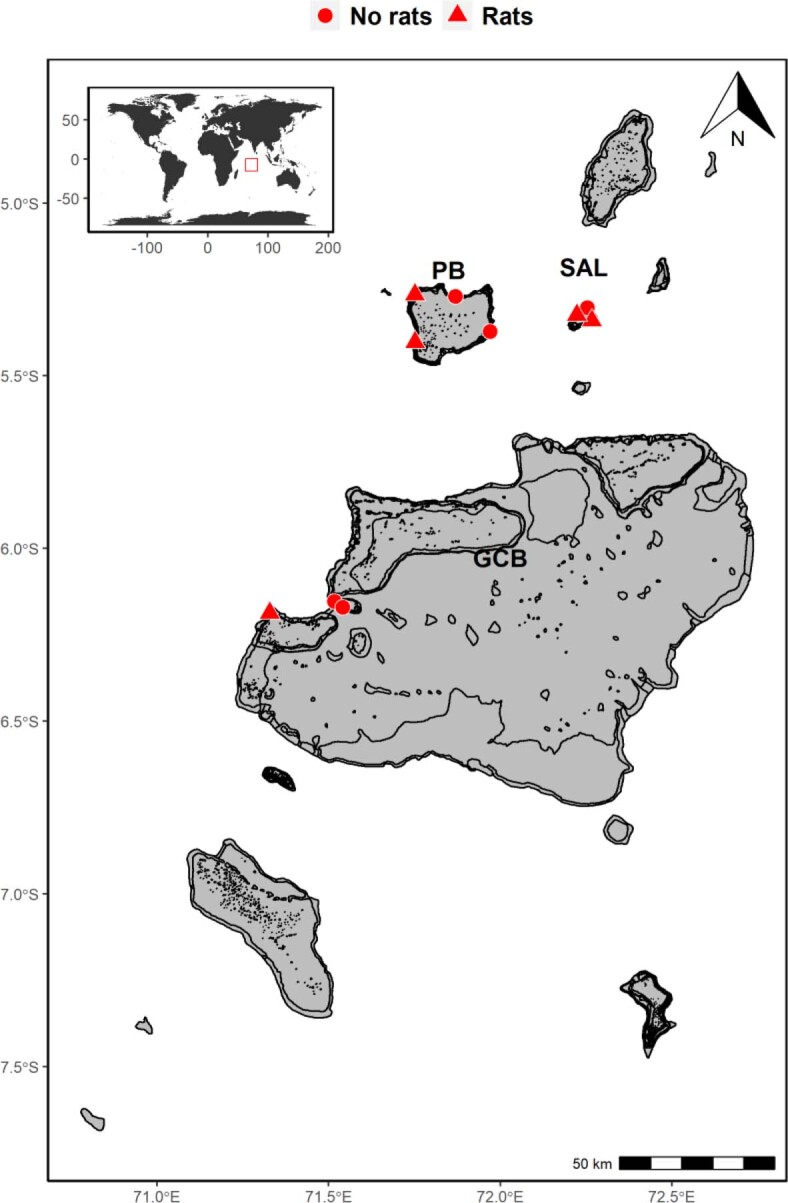
The location of study sites around the Chagos Archipelago. The location of the Chagos Archipelago in the Indian ocean is shown in the inset. Surveys were conducted around three atolls: Peros Banhos (PB), Salomon (SAL) and the Great Chagos Bank (GCB). Points represent the locations of the 10 reefs where surveys were conducted.

We studied ten islands (*n* = 5 rat-free islands, *n* = 5 rat-infested islands, Extended Data Fig. 1) across three atolls within the Chagos Archipelago, where seabird densities on rat-free islands are up to 720 times higher, and the nitrogen input provided by seabirds is 251 times greater, than around rat-infested islands^22^. We calculated the area of 60 damselfish territories (*n* = 30 around islands with seabirds, *n* = 30 around islands with rats), and recorded the aggressive behaviour of 57 of these 60 individuals (*n* = 28 around islands with seabirds, *n* = 29 around islands with rats). *P. lacrymatus* is a herbivorous farmer damselfish that aggressively defends small solitary territories^30^, allowing territorial behaviour to be accurately linked to an individual’s nutritional resources.

## Nutritional resources

**Fig. 1:**
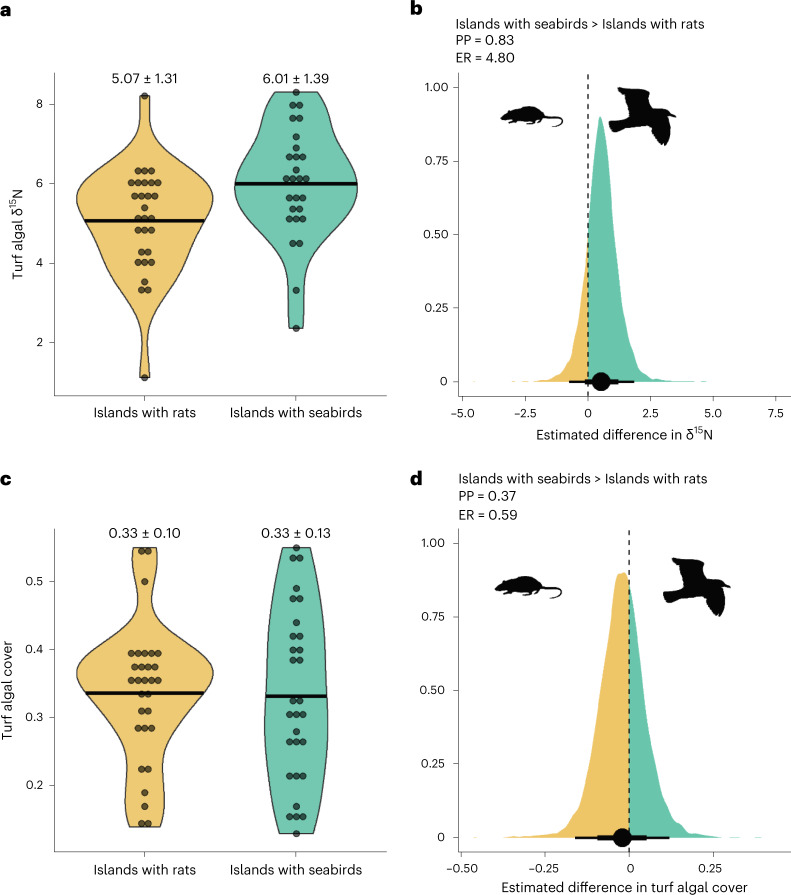
Turf algal δ^15^N and turf algal cover around islands with seabirds and islands with invasive rats within the Chagos Archipelago. **a**,**c**, Each point on the violin plots represents a single *P. lacrymatus* territory. Black bars show the mean estimates for turf algal δ^15^N (**a**, *n* = 27 around islands with seabirds, *n* = 29 around islands with rats) and for turf algal cover (**c**, *n* = 20 around islands with seabirds, *n* = 30 around islands with rats). Means ± s.d. are presented above each violin plot. The upper and lower bounds of the violin plots show the range of the raw data. **b**,**d**, Bayesian posterior densities show the effect of island invasion status on turf algal δ^15^N (**b**) and turf algal cover (**d**). Points are median estimates, with thick and thin lines representing 75% and 95% credible intervals, respectively. PPs, ERs and posterior densities in green show the extent to which nitrogen input (**b**) and turf algal cover (**d**) are higher around islands with seabirds. Rat and seabird graphics from PhyloPic.org under Public Domain Dedication 1.0 licences.

Seabird guano contains high levels of the ^15^N nitrogen isotope relative to ^14^N (δ^15^N). Elevated levels of δ^15^N act as an indicator of seabird-derived nutrient subsidies and have also been positively associated with reef fish growth rate^22^, suggesting that δ^15^N is also an indicator of resource quality. Within *P. lacrymatus* territories adjacent to islands with seabirds, there was a 0.83 posterior probability (PP) that turf algal δ^15^N was higher than around islands with rats (Fig. 1; slope: 0.53, 95% credible intervals: −0.46, 1.58, evidence ratio (ER): 4.80). The posterior probability for this effect is similar to previous estimates of elevated turf algal δ^15^N adjacent to islands with seabirds^22,25^. Given the absence of any additional human stressors around the study islands, this isotopic enrichment can be attributed directly to seabird nutrient subsidies in the absence of invasive rats.

There was no evidence for a difference in turf algal cover with island invasion status (slope: 0.02 (−0.13, 0.09), PP: 0.37, ER: 0.59; Fig. 1). While the absence of invasive rats does not appear to influence the amount of turf algae available to *P. lacrymatus* individuals, the nutritional enrichment of algae was higher within *P. lacrymatus* territories where seabird nutrient subsidies were present. Consequently, in the absence of invasive rats, *P. lacrymatus* achieve greater nutritional gain per unit of foraging effort.

## Territory size

**Fig. 2:**
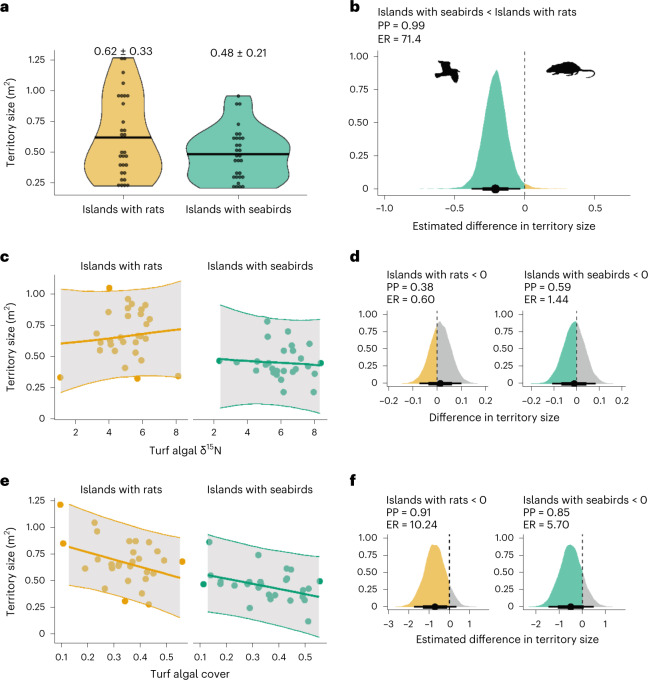
Variation in *P. lacrymatus* territory size between and within island invasion status type with turf algal δ^15^N and turf algal cover within the Chagos Archipelago. **a**, Raw data showing territory size estimates for *P. lacrymatus* individuals (*n* = 30 around islands with seabirds, *n* = 30 around islands with rats). Each point represents a single *P. lacrymatus* territory. Black bars show the mean estimates for *P. lacrymatus* territory size, and mean ± s.d. are also presented above each violin plot. The upper and lower bounds of the violin plots show the range of the raw data. **b**, Bayesian posterior density showing the effect of island invasion status on *P. lacrymatus* territory size. Points are median estimates, with thick and thin lines representing 75% and 95% credible intervals, respectively. The PP, ER and posterior density in green show the extent to which *P. lacrymatus* territories are smaller around islands with seabirds. **c**,**e**, Relationships between turf algal cover (**e**), turf algal δ^15^N (**c**) and *P. lacrymatus* territory size within island invasion status type. Points are partialized residuals extracted from Bayesian models for each *P. lacrymatus* individual. Best fit lines are extracted from Bayesian model conditional effects, with grey shading indicating 75% quantiles around the mean estimate. **d**,**f**, Posterior density plots showing the strength of the relationships in **c** and **e**, respectively. Densities to the right of 0 indicate a positive relationship, while densities to the left of 0 indicate a negative relationship. Evidence ratios show how much more likely the observed relationship is present over the alternative (grey shading). Rat and seabird graphics from PhyloPic.org under Public Domain Dedication 1.0 licences.

**Extended Data Fig. 2:**
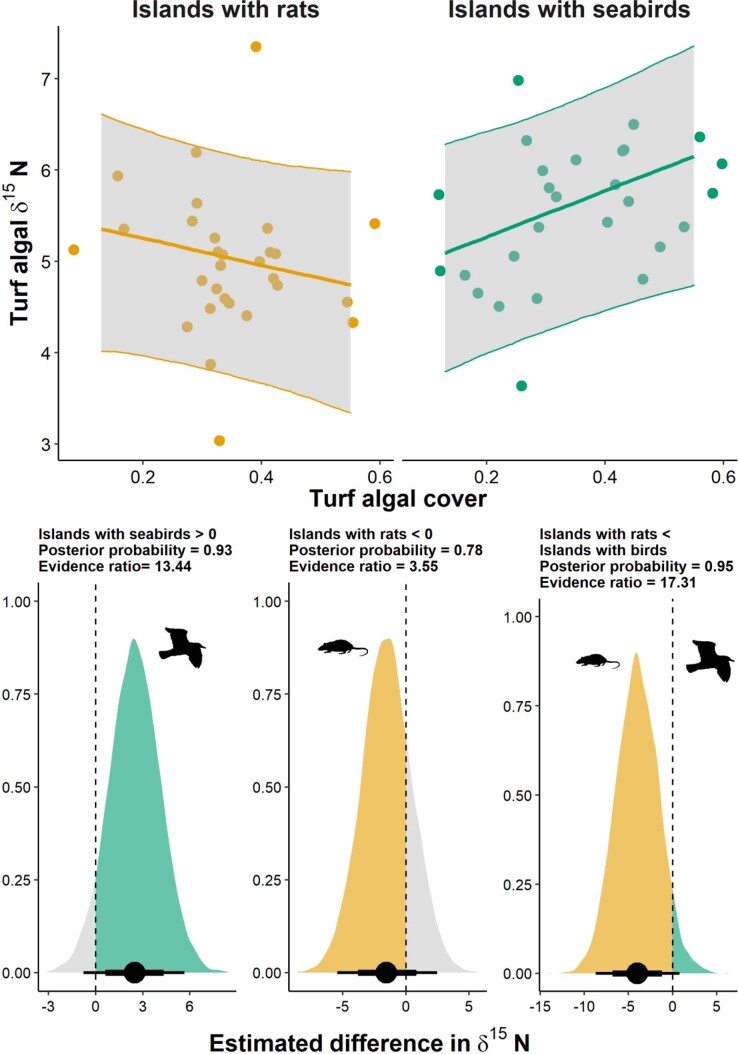
The relationship between turf algal cover and δ^15^N within *P. lacrymatus territories*. Top: Points represent partialized residuals extracted from Bayesian models for each *P. lacrymatus* individual around islands with seabirds (Green) and islands with rats (Yellow). Points are presented alongside best fit lines based on Bayesian model conditional effects, with grey shading indicating 75% quantiles. Bottom: Bayesian posterior densities from hypothesis tests. Posterior probabilities and evidence ratios show the extent to which 1) a positive relationship is supported around islands with seabirds (Left, green), 2) A negative relationship is supported around islands with rats (Middle, yellow), and 3) The relationship between turf algal cover and δ^15^N is different (that is, more negative) for *P. lacrymatus* territories around islands with rats compared to around islands with seabirds (Right, yellow).

The greater nutritional gain per unit foraging effort around islands with seabirds resulted in *P. lacrymatus* individuals holding smaller territories compared with individuals around islands with rats, with a posterior probability of 0.99 (Fig. 2; slope: −0.21 (−0.34, −0.07), ER: 74.47). The nutritional content of algal resources can shape a trade-off between territory size and quality, with smaller territories yielding higher-quality nutritional resources^31^. Where nutrient subsidies are present, individuals can meet short-term energetic demands faster through a higher nutritional gain per unit foraging effort, allowing individuals to hold smaller territories^7,32^. Where seabird nutrient subsidies are absent, *P. lacrymatus* individuals need to consume a greater amount of turf algae to maintain short-term energetic demands. As there was no difference in the amount of turf algae available across the study islands, these individuals need to hold larger territories to maximize nutritional gain^12^ (Fig. 2). This observation is also reflected in the association between turf algal cover and turf algal δ^15^N around islands with rats, such that territories with the highest turf algal cover had the lowest δ^15^N values (Extended Data Fig. 2 and Table 1). There are, therefore, broad-scale differences in *P. lacrymatus* territory size that, given the lack of variation in turf algal cover with invasion status, are most likely driven by the observed differences in turf algal δ^15^N, which is a direct consequence of the presence or absence of invasive rats.

The design of our study allows the relative roles of resource quantity and quality in driving territoriality within and between island invasion status types to be understood. Nutritional resources are predicted to influence territoriality until a point of ‘consumer saturation’, at which point the benefits of territoriality have been maximized^19^. *P. lacrymatus* territory size was inversely associated with turf algal cover, providing support for the food maintenance hypothesis^7,32^ with a PP of 0.85 for islands with seabirds and 0.91 for islands with rats (Fig. 2; slope: −0.50 (−1.31, 0.32), ER: 5.70 around islands with seabirds, slope: −0.71 (−1.59, 0.17), ER: 10.24 around islands with rats). Around islands with seabirds, δ^15^N appeared to be at a point of consumer saturation^19^ such that the energetic benefits of elevated δ^15^N in determining territory size were maximized and fine-scale variation in δ^15^N had no further effect on *P. lacrymatus* territory size (slope: −0.01 (−0.09, 0.07), PP: 0.59, ER: 1.44; Fig. 2). There was also no association between *P. lacrymatus* territory size and turf algal δ^15^N around islands with rats (slope: 0.01 (−0.06, 0.08), PP: 0.38, ER: 0.60). In the absence of nutrient subsidies where overall turf algal δ^15^N is low, slight variation in turf algal δ^15^N probably yields a lower energetic pay-off^18,19^ than variation in turf algal cover, indicating a trade-off between resource quality and quantity. As the evidence for both smaller territory sizes (PP = 0.99) and decreasing territory size with increasing turf algal cover ratio (PP = 0.91) for *P. lacrymatus* around islands with rats is relatively strong, resource quantity appears to be the primary determinant of *P. lacrymatus* territory size where nutrient subsidies are absent. These results offer strong support that nutrient disruption by an invasive terrestrial species has direct consequences on the territoriality of a native marine species.

## Aggression

**Fig. 3:**
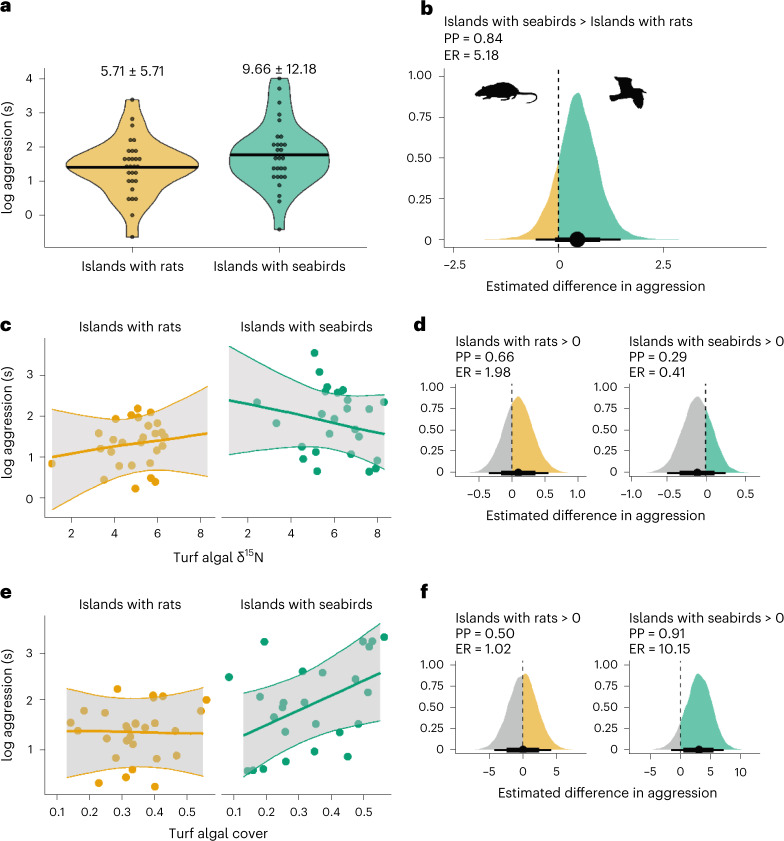
*P*. *lacrymatus* aggression between and within island invasion status type with turf algal δ^15^N and cover within the Chagos Archipelago. **a**, Raw aggression estimates for *P. lacrymatus* individuals (*n* = 28 around islands with seabirds, *n* = 29 around islands with rats). Each point represents a single *P. lacrymatus* territory. Black bars are mean estimates, and mean ± s.d. are also presented above each violin plot. The upper and lower bounds of the violin plots show the range of the raw data. **b**, Bayesian posterior density showing the effect of island invasion status on *P. lacrymatus* aggression. Points are median estimates, with lines representing 75% and 95% credible intervals, respectively. The PP, ER and posterior density in green show the extent to which *P. lacrymatus* aggression is higher around islands with seabirds. **c**,**e**, Relationships between turf algal cover (**e**), turf algal δ^15^N (**c**) and *P. lacrymatus* aggression within island invasion status type. Points are partialized residuals extracted from Bayesian models, with best fit lines extracted from Bayesian model conditional effects. Grey shading indicates 75% quantiles around the mean estimate. **d**,**f**, Posterior density plots showing the strength of the relationships in **c** and **e**, respectively. Densities to the right of 0 indicate a positive relationship, while densities to the left of 0 indicate a negative relationship. Evidence ratios show how much more likely the observed relationship is present over the alternative (grey shading). Rat and seabird graphics from PhyloPic.org under Public Domain Dedication 1.0 licences.

The higher nutritional gain per unit foraging effort available to *P. lacrymatus* adjacent to islands with seabirds provides individuals with the energy to invest more time in aggressive territory defence than individuals adjacent to islands with invasive rats, with a PP of 0.85 (Fig. 3; slope: 0.47 (−0.35, 1.28), ER = 5.47)^9,33^. Furthermore, this broad-scale effect is driven by fine-scale variation in turf algal cover and δ^15^N in *P. lacrymatus* territories within island invasion status. Adjacent to islands with seabirds, the overall elevated turf algal δ^15^N has maximized the benefits of turf algal δ^15^N for *P. lacrymatus* aggression, resulting in no relationship between fine-scale variation in δ^15^N and aggression (Fig. 3; slope: −0.11 (−0.43, 0.21), PP: 0.29, ER: 0.41). Variation in aggression is instead driven by turf algal cover within each *P. lacrymatus* individual’s territory (Fig. 3; slope: 2.97 (−0.72, 6.43), PP: 0.91, ER: 10.15). As turf algal cover increases, the value of the territory increases, and the pay-off of being aggressive is higher than remaining passive in the presence of nutrient subsidies^19,34^. It is also possible that variation in aggression is a consequence of *P. lacrymatus* having evolved an inherently lower threshold for aggression in the absence of invasive rats. Aggression is heritable^35^, and the invasion status of the study islands has been consistent since the introduction of black rats in the 1700s^36^.The short larval duration of *P*. *lacrymatus* (~23 days)^37^ and the prevalence of self-recruitment among reef fish^38^ suggest it is plausible that *P. lacrymatus* individuals adjacent to islands with seabirds only recruit to islands with seabirds and vice-versa. Both the evolutionary history of the economics of territoriality and the behavioural history of individuals^39^ can have important consequences for the persistence of species^11^.

**Fig. 4:**
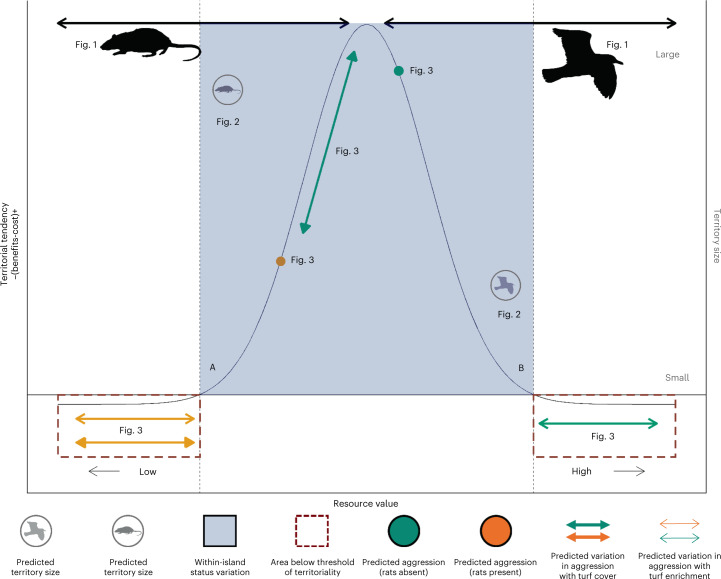
Threshold model of economic defendability with results for damselfish territoriality in the presence and absence of seabird nutrient subsidies. Territoriality is predicted to occur where the benefits outweigh the cost (shaded blue area). Below the threshold of territoriality, there is predicted to be no relationship between resource value and territoriality (dashed red boxes). The presence of nutrient subsidies around islands with seabirds is predicted to increase resource value to damselfish, resulting in higher levels of aggression (green point) than around islands with invasive rats (orange point). An inverse relationship between resource value and territory size (secondary *y* axis) is also predicted such that territories of higher resource value, that is, those around islands with seabirds, will be smaller (circular bird icon) than territories with lower resource value, that is, around islands with invasive rats (circular rat icon). Around islands with rats, resource value is low, and variation in turf algal cover and turf algal δ^15^N is not enough to place *P. lacrymatus* individuals above the threshold of territoriality (orange arrows). Around islands with seabirds, elevated δ^15^N is high, placing *P. lacrymatus* territories beyond the threshold of territoriality (green arrow with open arrowhead). Variation in aggression within reefs adjacent to islands with seabirds is instead driven by variation in turf algal cover (green arrow with closed arrowhead). Rat and seabird graphics from PhyloPic.org under Public Domain Dedication 1.0 licences.

In the presence of invasive rats and the subsequent absence of nutrient subsidies, the costs of aggression (energetic expenditure) appear to outweigh the benefits (energetic gain) even where turf algal cover is high. The resource value of these *P. lacrymatus* territories primarily remains below the threshold where aggression would be beneficial, resulting in no association between either turf algal cover (slope: 0.00 (−3.48, 3.45), PP: 0.50, ER: 1.02) or δ^15^N (slope: 0.09 (−0.26, 0.45), PP: 0.66, ER: 1.98; Figs. 3 and 4). The disruption of nutrients by invasive black rats has therefore reduced the aggressive tendencies of *P. lacrymatus* individuals (Fig. 4).

As farming damselfish, *P. lacrymatus* have the capacity to add nutrients to their algal farm, resulting in a higher productivity of algal turfs within *P. lacrymatus* territories compared with algal turfs outside the territories^40^. Around rat-infested islands, it is therefore possible that lower aggression by *P. lacrymatus* was traded off by an increase in farming behaviours to raise the productivity of algal resources within *P. lacrymatus* territories. However, as the focus of this study is on the specific role of seabird-derived nutrients, i.e. δ^15^N within algal resources, the impact of *P. lacrymatus* adding nutrients to their algal farms on our results and inferences is likely to be negligible. Similarly, seasonal variation in nutrient cycling that may impact the seabird-derived nutrient loads onto the coral reefs around our study sites may also be negligible. We controlled for environmental variation that could impact nutrient loads, such as weather conditions (see Methods). Furthermore, as there are nesting seabirds on rat-free islands year-round, including species that are large contributors to the seabird-derived nutrient budget^41^, seasonal variation in seabird densities is also unlikely to have a substantial impact on the observed differences in *P. lacrymatus* behaviour in the presence and absence of seabird-derived nutritional subsidies.

## Ecosystem-level consequences

**Extended Data Fig. 3:**
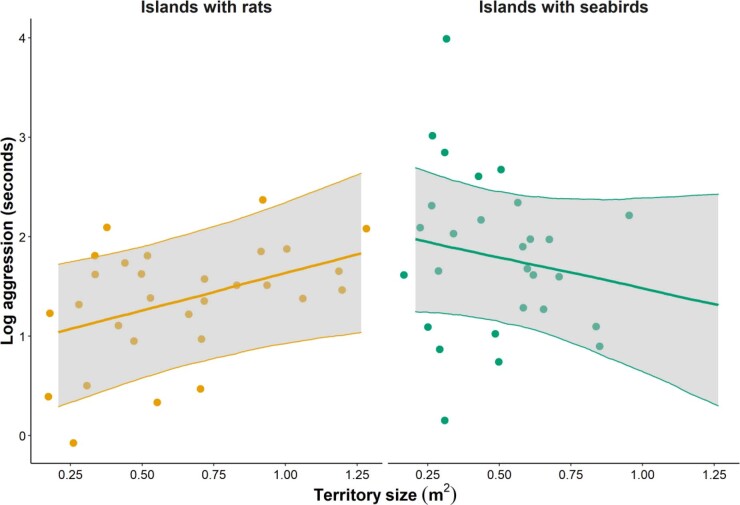
The influence of *P. lacrymatus* territory size on aggression around islands with seabirds (Green) and islands with rats (Yellow). Points represent partialized residuals extracted from Bayesian models for each *P. lacrymatus*. Best fit lines are extracted from Bayesian model conditional effects, with grey shading indicating 75% quantiles around the mean estimate.

The impact of invasive species in terrestrial ecosystems on the territoriality of reef fish (Fig. 4, Extended Data Fig. 3, and Supplementary results and discussion) could have broader community-level consequences for coral reef ecosystems. *P. lacrymatus* individuals adjacent to rat-infested islands display lower growth rates than individuals with access to seabird nutrient subsidies^22^. This has important consequences for fish biomass and productivity, with faster-growing, more aggressive fish adjacent to rat-free islands probably contributing to increased ecosystem function in the presence of seabird nutrient subsidies^22,29^.

**Extended Data Fig. 4:**
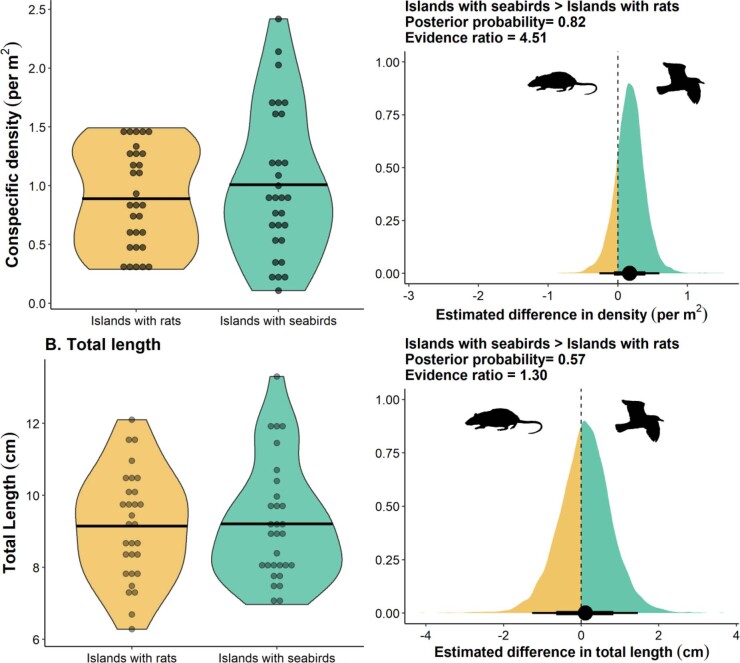
*P. lacrymatus* density and focal individual total length around islands with seabirds and islands with invasive rats within the Chagos Archipelago. Each point on the violin plots (left) represents a single *P. lacrymatus* territory. Mean estimates for conspecific density (A, left) around islands with seabirds (n = 30) and islands with rats (n = 30), and for focal individual total length (B, left) around islands with seabirds (n = 30) and islands with rats (n = 30) are represented by black bars. Posterior densities (Right) in green show the extent to which the following hypotheses are supported.: 1. Conspecific density (A, right) is higher around islands with seabirds and 2. Focal individual total length (B, right) is higher around islands with seabirds. Evidence ratios show how much more likely these hypotheses are supported over the alternative hypotheses. Rat and seabird graphics from PhyloPic.org under Public Domain Dedication 1.0 licenses.

**Extended Data Fig. 5:**
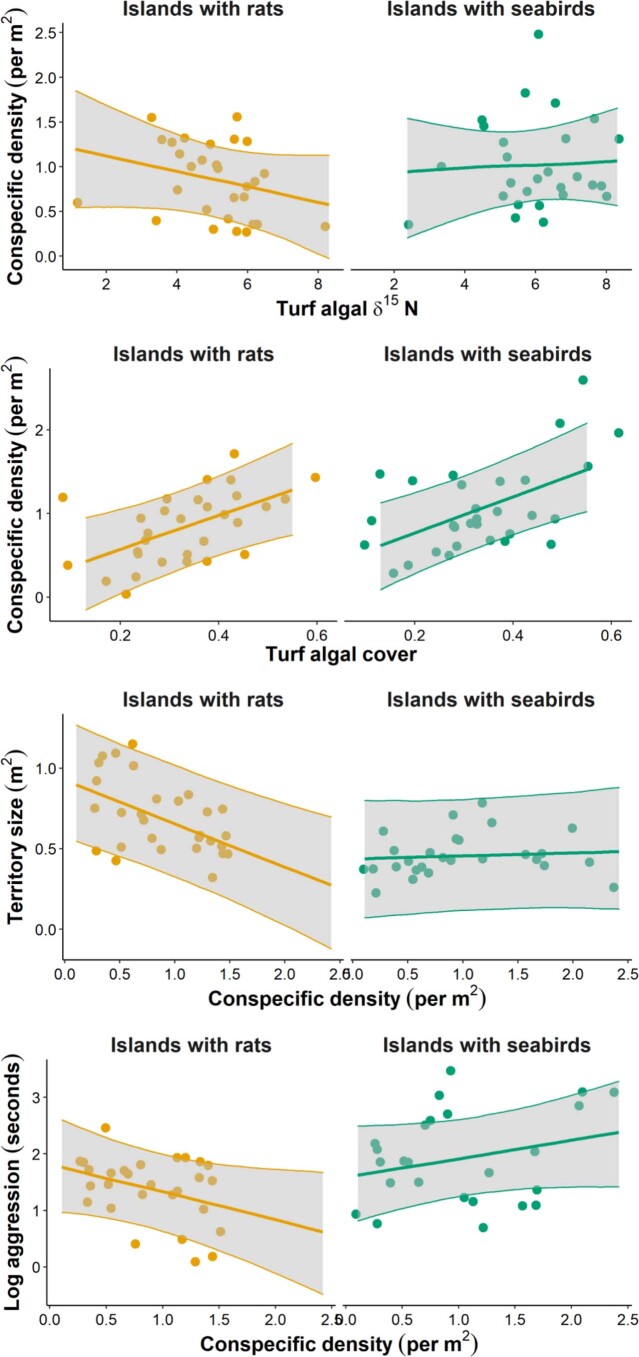
*P. lacrymatus* conspecific density, nutritional resources, and *P. lacrymatus* territoriality. Points represent partialized residuals extracted from Bayesian models for each *P. lacrymatus* individual around islands with seabirds (Green) and islands with rats (Yellow). Best fit lines are extracted from Bayesian model conditional effects, with grey shading indicating 75% quantiles 75% quantiles around the mean estimate.

**Extended Data Fig. 6:**
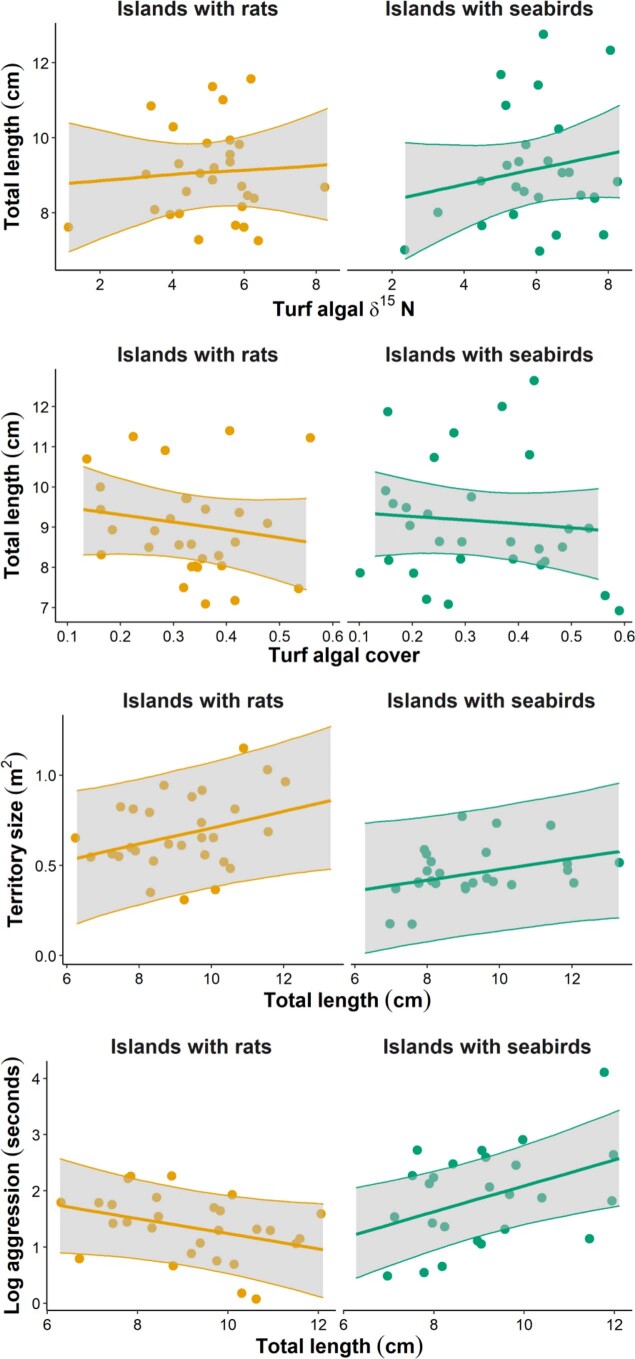
*P. lacrymatus* total length, nutritional resources, and *P. lacrymatus* territoriality. Points represent partialized residuals extracted from Bayesian models for each *P. lacrymatus* individual around islands with seabirds (Green) and islands with rats (Yellow). Best fit lines are extracted from Bayesian model conditional effects, with grey shading indicating 75% quantiles 75% quantiles around the mean estimate.

As a functionally important reef fish^42^, the aggressive nature of *P. lacrymatus* and other territorial damselfish can influence the spatial and social organization of reef fish communities. For example, space use and foraging areas of butterflyfish (Chaetodontidae) are influenced by high levels of interspecific aggression from damselfish^43^. The density of surgeonfishes, such as *Acanthurus coeruleus*, is also negatively associated with territorial damselfish density, and damselfish density is a predictor of surgeonfish social mode^44^. Furthermore, by driving variation in *P. lacrymatus* territory size, the disruption of nutrients by invasive rats also affects the spatial organization of territorial damselfish (Extended Data Figs. 4–6 and Table 2), which may have broader impacts on the reef community. *P. lacrymatus* have the capacity to influence the composition of both algal and coral communities^45^. Damselfish territories are areas of high algal productivity^46^, but also act as a refuge for some species of coral^46^ while contributing to the mortality of others^47^. The role of invasive rats in altering *P. lacrymatus* territory size could therefore have indirect consequences for coral growth, community composition and resilience.

We have provided new insights into how the disruption of cross-ecosystem nutrient subsidies by a terrestrial invasive species can influence territorial behaviour in a reef fish. Given the global decline in the movement of nutrients across ecosystems, understanding the proximate ecological implications of nutritional declines, such as behavioural responses of affected organisms, is a fundamental step in understanding the ultimate consequences of declining nutrient subsidies on species persistence^11^. The presence of invasive rats impacts reef fish species interactions via changes in territorial behaviour, impacting benthic and reef fish community composition and biodiversity, and subsequently ecosystem function and resilience^25,29^. Rat eradication therefore has the potential to have multiple cross-ecosystem benefits, from restoring territoriality in individual reef fish to the subsequent bottom-up effects on populations, communities and ecosystems.

## Methods

### Site and study species

We completed all data collection between 14 April and 6 May 2021 around the remote northern islands of the Chagos archipelago, which is part of a large no-take marine protected area in the Indian Ocean^48^. The northern reefs of the archipelago are some of the most pristine in the world, characterized by extremely high fish biomass^22^. The study area is free of local human stressors, except for invasive rats^36^. In total we surveyed 10 islands across 3 atolls (Extended Data Fig. 1). Five of the islands are infested with black rats (*Rattus rattus*) and 5 have high seabird densities and no rat infestations. Seabird densities around rat-free islands have been shown to be 760 times higher than around rat-infested islands^22^. Otherwise, all islands are similar in terms of environment and size^36,48^. Surveys addressing the impact of seabird-derived nutrient subsidies on both terrestrial and marine ecosystems have been conducted around all of the study islands every year between March and April since 2015. All work was conducted on the inward side of the islands close to shore. Survey sites were therefore selected to reduce the impact of variables such as seasonal variation in water and weather conditions.

*Plectroglyphidodon lacrymatus* is categorized as an extensive herbivorous farmer, with both macroalgae and turf algae found within their algal farms^30^. *P. lacrymatus* has also been used in previous studies to quantify nitrogen isotope signals in the presence and absence of invasive rats across the Chagos Archipelago^22^. Specifically, *P. lacrymatus* around rat-free islands have also been shown to have an elevated nitrogen signature (δ^15^N) and faster growth rate compared with individuals around rat-infested islands^22^.

### Behavioural observations

At each of the 10 islands, we randomly selected 6 focal *P. lacrymatus* individuals from around sites that were surveyed in 2015^22^ and in 2018^27^. These survey sites were marked by a GPS in 2015, and the distance of these sites from the island shore was also recorded^22^. We placed a Go Pro camera in the vicinity of the territories of the focal *P. lacrymatus* individuals. Cameras were deployed for 20 min, with the first 5 min discarded as an acclimation period^49^ to ensure the focal individual was not influenced by the presence of observers or the camera. After 20 min, cameras were collected and videos analysed in the laboratory. We used a continuous sampling approach when conducting video analyses, recording behaviours and the time at which behaviour changed. Videos were recorded between approximately 9 am and 3 pm. While there is evidence that *P. lacrymatus* foraging behaviour varies with the time of day^50^, we considered population-level behaviour in our analyses, and recording videos across a 6 h time period allowed us to capture this natural variation. Subsequently, we were able to determine whether differences in aggressive behaviour are present as a consequence of the presence/absence of nutritional subsidies while controlling for natural variation in foraging behaviour, which could in turn result in natural variation in aggressive tendencies.

We recorded all *P. lacrymatus* aggressive interactions from the behavioural videos using the BORIS software^51^. Behaviours associated with aggressive interactions included attacking in short accelerated swimming movements, biting and butting^52^. For all aggressive interactions, the encountered species was recorded as either a conspecific or heterospecific. We noted both the number of aggressive interactions and the total length of all aggressive interactions for each individual. We were unable to determine the sex of focal individuals as there is no sexual dimorphism for this species, and we did not collect the focal individuals to determine sex via dissection. While it is possible that there could be an effect of sex on territorial behaviour, this would probably only be the case for territoriality associated with mating, specifically egg guarding, and there was no evidence of such behaviour in any of our videos. All behavioural videos are available via Figshare (10.6084/m9.figshare.21089770).

### Territory mapping

We mapped the territory of a single individual present at the location of each of the six Go Pro cameras placed for behavioural studies at each island using methods similar to those in ref. ^30^. We placed a camera on a stand 1 m above the territory of the individual such that the camera had a field of view of 2 × 2 m. A 25 cm scale was visible within the field of view of the camera. Cameras were left for 20 min, with the first 9 min then discarded as an acclimation period^49^. To calculate territory size, we took 21 screengrabs of the footage approximately every 30 s across a 10 min period. We then imported these screengrabs into ImageJ to record the position of the focal individual. We set the frame of the images as *X*, *Y* axes and recorded the individual positions as Cartesian coordinates. We then plotted the Cartesian coordinate positions and calculated the minimum convex polygon of the points for each focal damselfish to estimate territory area.

We also used the territory camera footage to count the number of neighbouring *P. lacrymatus* individuals to estimate *P. lacrymatus* density for each individual patch surveyed within each island. The total length of each focal individual was also estimated from the territory camera footage.

For two of the islands (one rat-invested island and one island without rats, and a total of 12 *P. lacrymatus* individuals), territory cameras could not be used due to high currents and wave surge. Instead, we used Go Pro cameras on a photo time-lapse (one photo per second) to take photos of the focal damselfish territory, with a 0.5 × 0.5 m quadrat placed within the territory as a size reference. The photos were then imported into the software programme Agisoft and used to create three-dimensional (3D) models of the focal individual territories. To estimate territory size for these individuals, we used the behavioural observation video to mark the location of focal individuals every 30 s for 10 min as above, then cross referenced these locations with a screengrab of the 3D model in ImageJ using the frame of the image as *X*, *Y* axes as above. To estimate the total length of these individuals, we photographed each territory from above, with a 0.5 × 0.5 m quadrat as a size reference, ensuring the focal individual was also within the frame of the photo. These images were then imported into ImageJ and the size of each focal individual was estimated.

### Benthic composition

Benthic surveys were conducted using the territory mapping video footage. We took a screengrab of the territory area from the videos and overlaid 100 points onto this image. We recorded the benthos under each of these points to quantify the abundance and percentage cover of algae (turf and macro) within each territory. For the 12 individuals for which territory cameras could not be used, we utilized the 3D models to conduct the territory benthic surveys using the same methods as above.

### Isotope sampling

Following behavioural observations and territory mapping, we collected turf algae and macroalgae if present (*Halimeda* spp.) from within the territory of each focal individual. All algal samples were dried at 60 °C for 24 h or until dry, ahead of nitrogen stable isotope analysis. Dried samples were washed in a 10% hydrochloric acid solution to remove any contaminants and calcareous matter, and then centrifuged at 1,008 × *g* (3,000 r.p.m.) for 6 min. Samples were washed thoroughly with distilled water between centrifuge cycles. In total, samples were centrifuged four times. Samples were then dried for a final time. Isotope samples were then analysed at Lancaster University (UK). Samples were combusted using an Elementar Vario MICRO cube elemental analyser before being analysed in an Isoprime 100 isotope ratio mass spectrometer. Samples were analysed with the two international standards IAEA 600 and USGS 41, and a random subset of samples were run in triplicates to ensure readings were accurate. From the analysis, we extracted values for the ratios of the nitrogen isotope N15:14 (δ^15^N) for both turf and macroalgae.

### Statistical analysis

All our analyses were conducted using R v4.1.0^53^. We ran Bayesian models using the brms package^54^ implemented in STAN^55^. All Bayesian models were run for 5,000 iterations, with a warm-up of 1,000 iterations over four chains. We used weakly informative normal priors for all models^56^ and included a nested random intercept for atoll and the islands within each atoll to account for spatial non-independence. As there was no ecological basis to assume variation in slopes between the different islands within each atoll, we did not include random slopes in the models^22,27^. All models assumed a normal likelihood based on distribution plots for each response variable. To check model fit and convergence, we used trace-plots, graphical posterior predictive checks, effective sample sizes and the Gelman-Ruban convergence diagnostic (R-hat)^57^. We log-transformed the time invested in aggression to improve model convergence and remove divergent transitions that were present when we ran the model on untransformed data. A total of 5 models had up to 10 divergent transitions. However, all models had effective sample size values of over 1,000 and R-hat values of less than 1.01, indicating that the Markov) chains had converged well^58,59^. To check for heavily weighted influential data points in our models, we used Pareto-smoothed importance-sampling leave-one-out cross-validation (PSIS_LOO). Pareto-k values of over 0.7 are considered highly influential^59^. Where we had pareto-k values of over 0.7, we ran the models with and without the highly influential data points and compared the posterior distributions. We also extracted the conditional effects and partialized residuals from all models. We then used the conditional effects and partialized residuals to determine mean estimates and 75% uncertainty intervals of the posterior predictive distributions. All our models comparing between island invasion status therefore had the following basic structure:

$${\mathrm{Response}}\,{\mathrm{variable}}\sim 0 + {{{\mathrm{Invasion}}}}\,{{{\mathrm{status}}}} + \left( {1|{{{\mathrm{Atoll}}}}/{{{\mathrm{Island}}}}} \right)$$

Our models comparing aspects of nutritional resources and *P. lacrymatus* territoriality within each island invasion status type had the following basic structure:

$$\begin{array}{l}{\mathrm{Response}}\,{\mathrm{variable}}\sim 0 + {\mathrm{Explanatory}}\,{\mathrm{variable}\left( s \right)}\\ \times {{{\mathrm{Invasion}}}}\,{{{\mathrm{status}}}} + \left( {1|{{{\mathrm{Atoll}}}}/{{{\mathrm{Island}}}}} \right)\end{array}$$

We then used hypothesis testing to test a priori hypotheses for our models. All hypothesis tests were one-way, and for each test we calculated: the PP to determine the probability to which our hypotheses were supported, and ER to show the extent to which the evidence that our hypotheses is supported is greater than an alternative hypothesis (Extended Data Tables 1 and 2). Evidence ratios are transformed posterior probability values such that $\mathrm{ER} = \frac{{\mathrm{PP}}}{{(1 - \mathrm{PP})}}$.

#### Nutritional resources

We compared turf algal δ^15^N within focal individual territories using a Bayesian model, with island invasion status as the explanatory variable. The following hypothesis was then tested, and posterior probabilities and evidence ratios calculated using nonlinear hypothesis testing: δ^15^N will be higher for *P. lacrymatus* territories around islands where rats are absent.

To compare the proportion of turf algae within focal individual territories between islands with no rats and islands with rats, we used a Bayesian model with island invasion status (rats absent/rats present) as the explanatory variable. We then used a nonlinear hypothesis test to test the a priori hypothesis that there is a higher proportion of turf algae around rat-infested islands.

We also ran a model to test for a relationship between resource quality (turf algal δ^15^N) and quantity (turf algal cover) within *P. lacrymatus* territories, with δ^15^N as the response variable. We used an interaction term between turf algal cover and island invasion status, as the relationship between resource quality and quantity could be variable between islands with seabirds and islands with rats. We then used nonlinear hypothesis tests to test the strength of the relationship between turf algal δ^15^N and cover around islands with seabirds and islands with rats, and to see whether the relationship was different between the two island types.

To see whether turf algal δ^15^N and cover were influenced by the distance of the territory to shore, we ran two models, with an interaction term between the distances from the GPS marked survey sites to the shore and invasion status, and turf algal δ^15^N or cover as the response variable. We then tested the following a priori hypothesis for both models: Turf algal δ^15^N/cover will be higher around sites closest to shore for islands with seabirds.

#### Territory size and aggression between island types

We tested the effect of island invasion status on *P. lacrymatus* territory size, time spent on aggression, conspecific density and focal individual total length using Bayesian models, with island invasion status (rats absent/rats present) as the explanatory variable. The length of aggressive interactions was log-transformed to improve model fit. We performed hypothesis tests for four a priori hypotheses:

1. *P. lacrymatus* territory sizes will be smaller around islands where rats are absent.
2. *P. lacrymatus* aggression (in terms of the length of aggressive interactions) will be higher around islands where rats are absent.
3. *P. lacrymatus* densities will be higher around islands where rats are absent.
4. *P. lacrymatus* total length will be higher around islands where rats are absent.

We ran a Bayesian model to test the relative roles of resource quality and quantity on *P. lacrymatus* territory size. We ran two models, one with resource quantity (turf algal cover) as the explanatory variable, and the other with resource quality (turf algal δ^15^N) as the explanatory variable. We included an interaction term between the explanatory variable and island invasion status. We then tested two a priori hypotheses from each of the two models: Around islands with seabirds (1) and islands with rats (2), territories with a higher turf algal δ^15^N/cover will be smaller.

#### Territory size and aggression within island type

For both aggressive behaviour and territory size, we considered the influence of turf algal δ^15^N, turf algal cover, conspecific density and focal individual total length within islands with seabirds and islands with rats, including an interaction term between each explanatory variable and island invasion status. We considered multiple variables to determine the strength of the relationship between territoriality and nutritional resources while controlling for additional biotic variables (conspecific density and total length). We ran two separate models for territory size and aggression, with all four of the above explanatory variables included in each model. We then ran a priori hypothesis tests to determine the strength of relationships between territory size and aggressive behaviour and each of the four explanatory variables.

In addition, we looked at the influence of territory size on aggression with an additional Bayesian model, with aggression as the response variable and territory size as the explanatory variable, with an interaction term between the explanatory variable and island invasion status also included. We used nonlinear hypothesis tests on this model to test the following a priori hypotheses: Around islands with seabirds (1) and islands with rats (2), aggression will be highest for *P. lacrymatus* individuals in the smallest territories, and this relationship will be weaker around islands with rats (3).

### Reporting summary

Further information on research design is available in the Nature Portfolio Reporting Summary linked to this article.

### Supplementary information

Supplementary Information Supplementary methods, results and discussion and extended data legends.

Reporting Summary.

Supplementary Data BORIS file containing the raw analysis of behavioural videos.

## Figures and Tables

**Extended Data Table 1:**
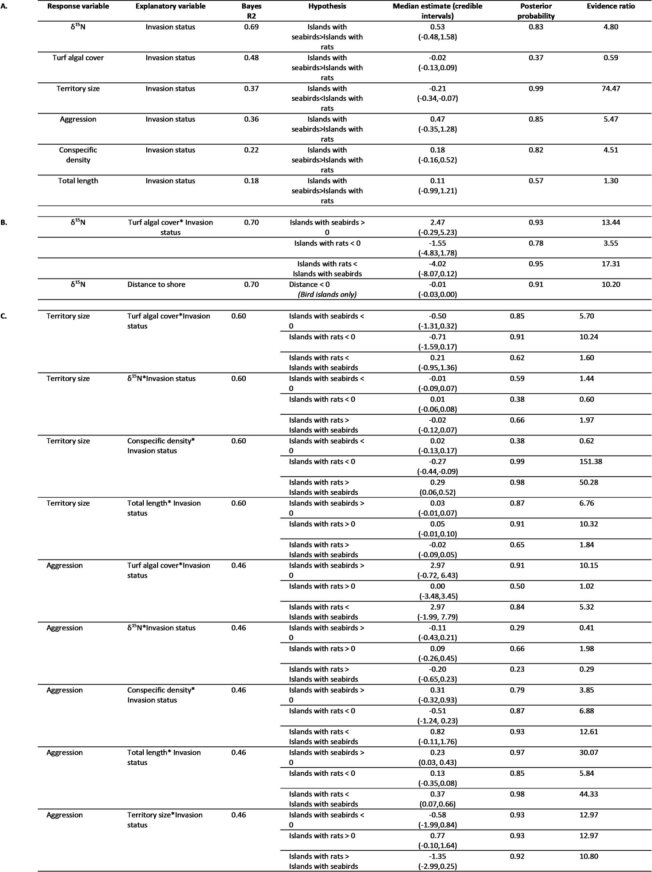
Summary of Bayesian models and hypothesis tests

A. Summary of models testing the role of island invasion status on nutrients, *P. lacrymatus* territory size, *P. lacrymatus* aggression, *P. lacrymatus* conspecific density and *P. lacrymatus* total length; B. Summary of models testing the relationship between turf algal δ^15^N and turf algal cover within *P. lacrymatus* territories; and C. Summary of models testing the relationship between nutrients (turf algal δ^15^N and cover) and two biotic variables (conspecific density and focal individual total length) on *P. lacrymatus* territory size and aggression. All models included a nested random intercept for island within each of the three study atolls.

**Extended Data Table 2:**
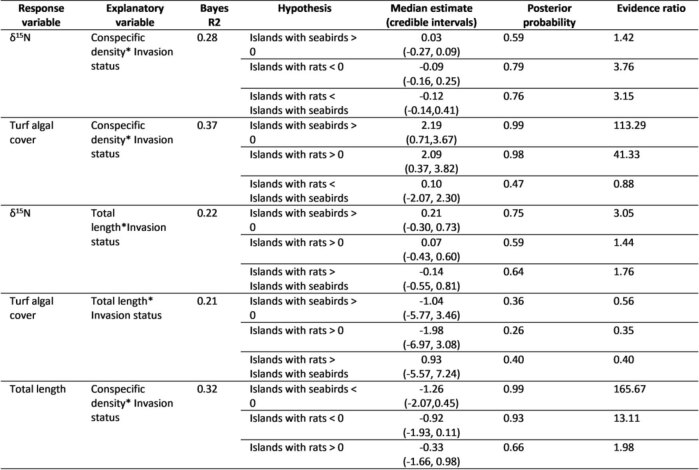
Summary of supplementary Bayesian models and hypothesis tests

Summary of models controlling for the role of nutrients (turf algal δ^15^N and percentage cover) on *P. lacrymatus* conspecific density and total length. All models included a nested random intercept for island within each of the three study atolls.

## Data Availability

The dataset associated with this work will made publicly available through the Figshare repository at 10.6084/m9.figshare.21089770

## Code availability

The code associated with this work will be made publicly available through the Figshare repository at 10.6084/m9.figshare.21089770
