# Supplementary material for: Terrestrial invasive species alter marine vertebrate behaviour
Source: Nat Ecol Evol. 2023 Jan 5;7(1):82–91. doi: 10.1038/s41559-022-01931-8 (PMC9834043; doi:10.1038/s41559-022-01931-8)
Supplement: Supplementary file 1 — Supplementary methods, results and discussion and extended data legends. [file 41559_2022_1931_MOESM1_ESM.pdf]

nature ecology & evolution

Article https://doi.org/10.1038/s41559-022-01931-8

Terrestrial invasive species alter marine vertebrate behaviour

In the format provided by the authors and unedited

Supplementary results and discussion

Results from all Bayesian models and hypothesis tests are presented in extended data Tables 1 and 2.

Nutritional resources

Around islands with seabirds, *P. lacrymatus* territories with high turf algal cover also contained turf algae with the highest $\delta^{15}N$ values (PP=0.93 (2.47 (-0.29,5.23)), extended data Fig. 1). The input of $\delta^{15}N$ from seabird nutrient subsidies occurs on a gradient, such that $\delta^{15}N$ is highest closer to shore[27].The presence of seabird nutrient subsidies has also been shown to enhance algal abundance[29]. *P. lacrymatus* territories closer to shore had higher $\delta^{15}N$ values (PP=0.91 evidence ratio=10.20), which is likely driving the positive association between turf algal $\delta^{15}N$ and turf algal cover around islands with seabirds.

Conspecific density and total length

Around islands with seabirds, *P. lacrymatus* density was 1.01 (±0.62) individuals per m^2 compared to 0.89 (±0.42) individuals per m^2 around rat-infested islands. Bayesian models and hypothesis tests showed an 0.82 posterior probability that conspecific density was higher around islands with seabirds than rat-infested islands (0.18 (-0.16, 0.52), extended data Fig. 2). The mean total length of focal individuals was 9.21 (±1.63) cm around islands with seabirds, compared to 9.15 (± 1.49) cm around islands with rats. There was no evidence to suggest that total length varied with islands invasion status (slope: 0.1 (-0.99, 1.21), PP: 0.57, extended data Fig. 2). Total length was instead a consequence of density dependence: *P. lacrymatus* individuals in areas of high conspecific densities had smaller total lengths around both islands with seabirds (slope: -1.26 (-2.07, -0.45), PP: 0.99, ER: 165.67) PP = 0.99 (-1.24 (-2.06, 0.41)) and around islands with rats (slope: -0.92 (-1.93, 0.11), PP: 0.93, ER: 13.11).

Territory size was negatively correlated with conspecific density around islands with rats, such that where conspecific focal individual territories were smaller at higher conspecific densities (extended data Fig. 5, slope: -0.27 (-0.44,0.09), PP:0.99, ER: 151.38 around islands with rats) but not around islands with seabirds (extended data Fig. 5, slope: 0.02 (-0.13,0.17), PP: 0.38, ER: 0.62). Around islands with seabirds, conspecific density was weakly positively correlated with length of aggression (slope: 0.31 (-0.32,0.93), PP: 0.79, ER: 3.85).
Conversely, around islands with rats, there was a negative relationship between conspecific density and time invested in aggression (slope: -0.51 (-1.24, 0.23) PP: 0.87, ER: 6.88).

Territory size was positively correlated with focal individual total length such that larger focal individuals held larger territories (extended data Fig. 6, slope: 0.02 (-0.01, 0.07), PP: 0.87, ER: 6.76 around islands with seabirds, slope: 0.05 (-0.01, 0.10), PP: 0.91, ER: 10.32 around islands with rats). As with conspecific density, the relationship between focal individual total length and aggression was also variable between islands with seabird and islands with rats. Specifically, there was a positive association between total length and aggression around islands with seabirds (slope: 0.23 (0.03,0.43), PP: 0.97, ER: 30.07) and a negative association around islands with rats (slope: 0.13 (-0.35, 0.08), PP: 0.85, ER: 5.84).

Supplementary methods

To determine if focal individual total length is driven by density dependence, we modelled P. lacrymatus total length as a response variable, with an interaction between islands invasion status and conspecific density as the explanatory variable. We then tested the following hypotheses with nonlinear hypothesis tests: Around islands with seabirds (1) and islands with rats (2) P. lacrymatus individuals will be smaller at higher conspecific densities.

Extended data Figure legends

Extended data Fig. 1: The location of study sites around the Chagos Archipelago. The location of the Chagos Archipelago in the Indian ocean is shown in the inset. Surveys were conducted around three atolls: Peros Banhos (PB), Salomon (SAL) and the Great Chagos Bank (GCB). Points represent the locations of the 10 reefs where surveys were conducted.

Extended data Fig. 2: The relationship between turf algal cover and δ^{15}N within *P. lacrymatus* territories. Top: Points represent partialized residuals extracted from Bayesian models for each *P. lacrymatus* individual around islands with seabirds (Green) and islands with rats (Yellow). Points are presented alongside best fit lines based on Bayesian model conditional effects, with grey shading indicating 75% quantiles. Bottom: Bayesian posterior densities from hypothesis tests. Posterior probabilities and evidence ratios show the extent to which 1) a positive relationship is supported around islands with seabirds (Left, green), 2) A negative relationship is supported around islands with rats (Middle, yellow), and 3) The relationship between turf algal cover and δ^{15}N is different (i.e., more negative) for *P. lacrymatus* territories around islands with rats compared to around islands with seabirds (Right, yellow).

Extended data Fig. 3: The influence of *P. lacrymatus* territory size on aggression around islands with seabirds (Green) and islands with rats (Yellow). Points represent partialized residuals extracted from Bayesian models for each *P. lacrymatus.* Best fit lines are extracted from Bayesian model conditional effects, with grey shading indicating 75% quantiles around the mean estimate.

Fig. 4: *P. lacrymatus* density and focal individual total length around islands with seabirds and islands with invasive rats within the Chagos Archipelago. Each point on the violin plots (left) represents a single *P. lacrymatus* territory. Mean estimates for conspecific density (A, left) around islands with seabirds (n=30) and islands with rats (n=30), and for focal individual total length (B, left) around islands with seabirds (n=30) and islands with rats (n=30) are represented by black bars. Posterior densities (Right) in green show the extent to which the following hypotheses are supported.: 1. Conspecific density (A, right) is higher around islands with seabirds and 2. Focal individual total length (B, right) is higher around islands with seabirds. Evidence ratios show how much more likely these hypotheses are supported over the alternative hypotheses. Rat and seabird graphics from

PhyloPic.org under Public Domain Dedication 1.0 licenses.

Extended data Fig.5: *P. lacrymatus* conspecific density, nutritional resources, and *P. lacrymatus* territoriality. Points represent partialized residuals extracted from Bayesian models for each *P. lacrymatus* individual around islands with seabirds (Green) and islands with rats (Yellow). Best fit lines are extracted from Bayesian model conditional effects, with grey shading indicating 75% quantiles around the mean estimate.

Extended data Fig.6: *P. lacrymatus* total length, nutritional resources, and *P. lacrymatus* territoriality. Points represent partialized residuals extracted from Bayesian models for each *P. lacrymatus* individual around islands with seabirds (Green) and islands with rats (Yellow). Best fit lines are extracted from Bayesian model conditional effects, with grey shading indicating 75% quantiles 75% quantiles around the mean estimate.

Extended data Table legends

Extended data Table 1: Summary of Bayesian models and hypothesis tests. A. Summary of models testing the role of island invasion status on nutrients, *P. lacrymatus* territory size, *P. lacrymatus* aggression, *P. lacrymatus* conspecific density and *P. lacrymatus* total length ; B. Summary of models testing the relationship between turf algal $\delta^{15}N$ and turf algal cover within *P. lacrymatus* territories; and C. Summary of models testing the relationship between nutrients (turf algal $\delta^{15}N$ and cover) and two biotic variables (conspecific density and focal individual total length) on *P. lacrymatus* territory size and aggression. All models included a nested random intercept for island within each of the three study atolls.

Extended data Table 2: Summary of supplementary Bayesian models and hypothesis tests. Summary of models controlling for the role of nutrients (turf algal $\delta^{15}N$ and percentage cover) on *P. lacrymatus* conspecific density and total length. All models included a nested random intercept for island within each of the three study atolls.
