# Supplementary material for: Terrestrial invasive species alter marine vertebrate behaviour
Source: Nat Ecol Evol. 2023 Jan 5;7(1):82–91. doi: 10.1038/s41559-022-01931-8 (PMC9834043; doi:10.1038/s41559-022-01931-8)
Supplement: Supplementary file 2 — Reporting Summary. [file 41559_2022_1931_MOESM2_ESM.pdf]

nature portfolio

Corresponding author(s): Rachel L. Gunn

Last updated by author(s): Apr 14, 2022

Reporting Summary

Nature Portfolio wishes to improve the reproducibility of the work that we publish. This form provides structure for consistency and transparency in reporting. For further information on Nature Portfolio policies, see our Editorial Policies and the Editorial Policy Checklist.

Statistics

For all statistical analyses, confirm that the following items are present in the figure legend, table legend, main text, or Methods section.

n/a	Confirmed	
☐	☒	The exact sample size (*n*) for each experimental group/condition, given as a discrete number and unit of measurement
☐	☒	A statement on whether measurements were taken from distinct samples or whether the same sample was measured repeatedly
☐	☒	The statistical test(s) used AND whether they are one- or two-sided<br>*Only common tests should be described solely by name; describe more complex techniques in the Methods section.*
☐	☒	A description of all covariates tested
☐	☒	A description of any assumptions or corrections, such as tests of normality and adjustment for multiple comparisons
☐	☒	A full description of the statistical parameters including central tendency (e.g. means) or other basic estimates (e.g. regression coefficient) AND variation (e.g. standard deviation) or associated estimates of uncertainty (e.g. confidence intervals)
☐	☒	For null hypothesis testing, the test statistic (e.g. *F*, *t*, *r*) with confidence intervals, effect sizes, degrees of freedom and *P* value noted<br>*Give P values as exact values whenever suitable.*
☐	☒	For Bayesian analysis, information on the choice of priors and Markov chain Monte Carlo settings
☐	☒	For hierarchical and complex designs, identification of the appropriate level for tests and full reporting of outcomes
☐	☒	Estimates of effect sizes (e.g. Cohen's *d*, Pearson's *r*), indicating how they were calculated

Our web collection on statistics for biologists contains articles on many of the points above.

Software and code

Policy information about availability of computer code

Data collection	No software was used for data collection
Data analysis	R version 4.1.0, BORIS software and ImageJ

For manuscripts utilizing custom algorithms or software that are central to the research but not yet described in published literature, software must be made available to editors and reviewers. We strongly encourage code deposition in a community repository (e.g. GitHub). See the Nature Portfolio guidelines for submitting code & software for further information.

Data

Policy information about availability of data

All manuscripts must include a data availability statement. This statement should provide the following information, where applicable:

- Accession codes, unique identifiers, or web links for publicly available datasets
- A description of any restrictions on data availability
- For clinical datasets or third party data, please ensure that the statement adheres to our policy

The data and code associated with this work will made publicly available through the Figshare repository. 10.6084/m9.figshare.19481393.

Field-specific reporting

Please select the one below that is the best fit for your research. If you are not sure, read the appropriate sections before making your selection.

☐ Life sciences ☐ Behavioural & social sciences ☒ Ecological, evolutionary & environmental sciences

For a reference copy of the document with all sections, see nature.com/documents/nr-reporting-summary-flat.pdf

Ecological, evolutionary & environmental sciences study design

All studies must disclose on these points even when the disclosure is negative.

Study description	The study aimed to assess and quantified how the presence of invasive rats on tropical islands can influence the territorial behaviour of herbivorous fish on adjacent coral reefs via the disruption of a seabird nutrient pathway due to invasive rats. This was achieved by quantifying and contrasting the aggression and territory size of 60 herbivorous damselfish (Plectroglyphidodon lacrymatus) between five rat-infested islands and five rat-free islands. Resource quality and quantity was also assessed by quantifying the levels of seabird derived nutrients (δ15N) in turf algae and the proportion of turf algae cover within P. lacrymatus territories, and contrasted with island invasion status.
Research sample	60 P. lacrymatus individuals were observed for this study. P. lacrymatus was selected as this species was previously used to identify nitrogen signals in reef fish around the study islands. P. lacrymatus is also known to be highly territorial and hold small territories, allowing territory size estimates to be made from stationary cameras. There are also preexisting growth rate estimates for this species around both rat-infested and rat-free islands All focal individuals were adults but sex was not determined. Turf algae was collected for isotope analyses as this is the primary food source of P. lacrymatus.
Sampling strategy	Coral reefs adjacent to 10 islands (5 rat-free, 5 rat-infested) were selected as these islands and coral reefs have been surveyed and monitored for the impact of invasive rats on terrestrial and marine ecosystems since 2015. 10 islands also allowed for replication of at least 3 islands across three atolls: Salomon (3 islands), Peros Banhos (4 islands) and Great Chagos Bank (3 islands). 6 P. lacrymatus individuals were observed around each island, to allow for sufficient replication within and between islands and atolls.
Data collection	Behavioural observations were done via the placement of GoPro cameras within P. lacrymatus territories. Cameras were placed by RLG and CEB, with RLG approving the final positioning of each camera. Turf algae was collected from directly beneath the territory camera frames (see Methods) by RLG and CEB, to ensure the turf algae sample was taken from within the focal P. lacrymatus territory. All video and statistical analyses were completed by RLG.
Timing and spatial scale	Data collection occurred between the 14th April and 6th May 2021, on a 6 day-on, 1-day off schedule. 10 islands were surveyed across three atolls in the Chagos Archipelago: Salomon (3 islands), Peros Banhos (4 islands) and Great Chagos Bank (3 islands).
Data exclusions	No data were excluded.
Reproducibility	Methodologies are described in full in the Methods, and the data and code used to produce the study findings are available through Figshare which will be accessible at publication (10.6084/m9.figshare.19481393). Behavioural videos are also available via Figshare through the link in the Methods. Turf algae percentage cover, and territory size estimates were collected for all fish territories. δ15N estimates could not be extracted for 4 turf algae samples (relevant to Figures 1A, 1B, 2C, 2D, 3C, 3D). Aggression estimates could not be made for 3 fish territories due to video error (relevant to Figure 3). All other attempts at replication were successful.
Randomization	We surveyed 5 rat-free islands and 5 rat-infested islands. All selected islands have been used to monitor the impact of invasive rats on adjacent coral reef ecosystems since 2015. We surveyed P. lacrymatus individuals around monitoring sites used to monitor reef fish and benthic composition in 2015 and 2018. Around each island, we left a minimum of 3 m between each focal P. lacrymatus territory.
Blinding	Blinding was not possible or relevant for the study sites, as site selection was based on survey locations from 2015 and 2018. For the behavioural video analyses, the invasion status of the islands was removed from the title of all video files as a form of blinding. Statistical analyses were not blinded.

Did the study involve field work? ☒ Yes ☐ No

Field work, collection and transport

Field conditions	Ocean conditions were calm for the duration of the study
Location	This study was conducted across 3 atolls within the Chagos Archipelago in the Indian Ocean (5º 50' S, 72º 00' E)
Access & import/export	All fieldwork was conducted, and samples collected, under research permit number 0002SE21, which was approved on 31st March 2021.
Disturbance	We sampled turf and macro algae for isotope analysis. All other data collection was observational.

Reporting for specific materials, systems and methods

We require information from authors about some types of materials, experimental systems and methods used in many studies. Here, indicate whether each material, system or method listed is relevant to your study. If you are not sure if a list item applies to your research, read the appropriate section before selecting a response.

Materials & experimental systems

n/a	Involved in the study
☒	☐ Antibodies
☒	☐ Eukaryotic cell lines
☒	☐ Palaeontology and archaeology
☐	☒ Animals and other organisms
☒	☐ Human research participants
☒	☐ Clinical data
☒	☐ Dual use research of concern

Methods

n/a	Involved in the study
☒	☐ ChIP-seq
☒	☐ Flow cytometry
☒	☐ MRI-based neuroimaging

Animals and other organisms

Policy information about studies involving animals; ARRIVE guidelines recommended for reporting animal research

Laboratory animals	No laboratory animals were used in this study
Wild animals	All work with wild animals was purely observational.
Field-collected samples	Turf and macro algae samples for isotope analyses were dried ahead of analysis.
Ethics oversight	This was an observational study and as such ethical approval was not required

Note that full information on the approval of the study protocol must also be provided in the manuscript.
